# Assessing and forecasting collective urban heat exposure with smart city digital twins

**DOI:** 10.1038/s41598-024-59228-8

**Published:** 2024-04-26

**Authors:** Xiyu Pan, Dimitris Mavrokapnidis, Hoang T. Ly, Neda Mohammadi, John E. Taylor

**Affiliations:** 1 School of Civil and Environmental Engineering, Georgia Institute of Technology, 790 Atlantic Dr NW, Atlanta, GA 30332 USA; 2 Faculty of the Built Environment, University College London, Gower St, London, WC1E 6BT UK

**Keywords:** Civil engineering, Environmental health

## Abstract

Due to population growth, climate change, and the urban heat island effect, heat exposure is becoming an important issue faced by urban built environments. Heat exposure assessment is a prerequisite for mitigation measures to reduce the impact of heat exposure. However, there is limited research on urban heat exposure assessment approaches that provides fine-scale spatiotemporal heat exposure information, integrated with meteorological status and human collective exposure as they move about in cities, to enable proactive heat exposure mitigation measures. Smart city digital twins (SCDTs) provide a new potential avenue for addressing this gap, enabling fine spatiotemporal scales, human-infrastructure interaction modeling, and predictive and decision support capabilities. This study aims to develop and test an SCDT for collective urban heat exposure assessment and forecasting. Meteorological sensors and computer vision techniques were implemented in Columbus, Georgia, to acquire temperature, humidity, and passersby count data. These data were then integrated into a collective temperature humidity index. A time-series prediction model and a crowd simulation were employed to predict future short-term heat exposures based on the data accumulated by this SCDT and to support heat exposure mitigation efforts. The results demonstrate the potential of SCDT to enhance public safety by providing city officials with a tool for discovering, predicting, and, ultimately, mitigating community exposure to extreme heat.

## Introduction

Heat exposure has the potential to result in acute illnesses with a high risk of death, such as heat stroke and heat exhaustion^1^. These risks are particularly severe for vulnerable populations, including the elderly, infants, and patients with cardiovascular or respiratory diseases^2^. Global climate change is exacerbating this risk. 37% of heat-related deaths can be attributed to climate change and rising temperatures induced by human activities^3^. Apart from the effects of climate change, the urban heat island (UHI) phenomenon ﻿imposes additional heat fluxes on the city population^4^. Evidence suggests that increased temperatures due to the UHI effect account for 40% of heat-related mortality in the UK^5^. The effect is likely to increase as the world’s population is projected to rise by 26% from 2019 to 2050, with more than 66% residing in urban areas^6^.

Heat exposure assessment studies have played an important role in the process of mitigating the impact of urban heat exposure on human health and well-being. Exposure assessment can identify high-risk spots for increased mitigation measures, including greening the built environment, shading and insulating buildings, and heat exposure alert systems for city residents^7–9^. In particular, mapping exposure to extreme heat can help urban planners identify priority areas for adaptation planning in the case of limited budgets and resources^10^ and locate populations with higher vulnerability to the impacts of extreme heat exposure^7,11^.

In order to effectively take action for heat mitigation, it is worth noting that heat exposure is dynamic in the spatiotemporal dimension. Heat exposure at the scale of tens of meters could manifest significant differences due to the dynamic nature and fine-scale heterogeneity of urban spaces. For instance, the urban microclimate is influenced by urban canyons and urban landscapes^12^. Heat exposures are also affected by human movement and time-activity patterns^13^. People may congregate at grocery stores, retail establishments, and other points of interest, which leads to potentially higher levels of heat exposure. However, most of the extant heat exposure assessment studies are at the macroscale (e.g., regions, cities) and mesoscale (e.g., census tracks, land planning units, cells defined by the authors). The number of studies at the micro-scale (e.g., locations, buildings) is limited. Thus, the effect of human movement on heat exposure has not been examined in depth.

Smart City Digital Twins (SCDTs) are an emerging approach to understanding and addressing urban challenges, such as unhealthy built environments, high energy consumption, and disaster responses, and may provide new insight into the heat exposure assessment^14^. Specifically, the SCDT is a digital model of cities that runs in real-time, utilizes high spatial–temporal resolution data, conducts prediction and what-if scenario tests, integrates multi-source information and human dynamics, visualizes human-infrastructure interactions, and thus enables proactive city management for making cities more comfortable and sustainable^15^. An SCDT for urban heat exposure assessment is expected to collect high spatiotemporal granularity meteorology and human behavior data, analyze and interpret the data continuously, and proactively provide references and decision-making support for heat exposure mitigation.

From the perspective of SCDTs, we built a novel framework for assessing heat exposure in cities in this work, which has three characteristics, including 1) having meteorology data streams at a fine spatiotemporal scale, 2) integrating human behavior information and meteorological status to capture collective heat exposure, and 3) enabling heat exposure prediction and supporting proactive heat mitigation decision-making. Specifically, a heat exposure assessment index was designed that takes into account both the heat stress and the number of people affected. Heat stress is calculated with temperature and humidity data collected by meteorological sensors, and the number of affected people is obtained through the detection and tracking of passersby at each location. The assessment index is monitored using continuous and real-time data streams on the temperature, the humidity, and the passersby count. The real-time data collection also enables the accumulation of the data. The historical data are fed into a Seasonal Autoregressive Integrated Moving Average (SARIMA) model to forecast future short-term heat exposure index values and support proactive heat exposure mitigation decisions. A simulation model of crowd movements during large social events is used to analyze “black swan” events and works as a complementary tool to enhance the performance of the heat exposure predictions and what-if scenario tests.

## Literature review

This research focuses on SCDTs, computer vision-based human behavior detection, and heat exposure assessment. Relevant prior research on SCDT and computer vision was reviewed, which provided the theoretical and technical foundation for this study. The prior efforts for heat exposure assessment were reviewed in this section to identify knowledge gaps and opportunities for developing better methods.

### Smart city digital twins

SCDT originated from research on digital twins. Digital twins are a set of digital models with high fidelity to simulate real conditions, combining the virtual with reality for closed-loop optimization in design, production, and service^16^. The specific implementation of digital twins is different in various industries and fields. For example, in the aerospace industry, a digital twin is considered as an ultrarealistic simulation of vehicles or systems, designed to mirror the operation of its actual counterpart. It is “multiphysics, multiscale, probabilistic … (and) uses the best available physical models, sensor updates, and fleet history” (p. 7)^17^. In the complex systems field, digital twins refer to “a set of virtual information constructs that fully describes a potential or actual physical manufactured product from the micro-atomic level to the macro-geometrical level”^18^, reflecting the multiscale attribute of complex systems.

The SCDT is a city digital model that leverages continuously collected spatial and temporal data from infrastructure and organizational systems to understand how the city is functioning^14^. Its specific use cases have included energy consumption management^19^, air quality monitoring^20^, disaster responses^21^, and urban walkability assessment^22^, among others. For example, Ham & Kim engaged citizens in creating the digital city model and raising public awareness of disaster risk by utilizing crowdsourced data^23^. Lin & Cheung integrated wireless sensor networks and building information models in order to monitor and control air quality in underground parking lots^20^.

One characteristic of SCDT studies is to highlight the integration of continuous data streams regarding infrastructure and humans^22,23^. For example, Fan et al. employed a social sensing technique to build an SCDT for disaster management, where community-based social media posts about infrastructure disruptions were obtained and analyzed to assess the impact of flooding^21^. Sensing technology is often used in SCDT studies to enable data streams with higher spatiotemporal resolution. For instance, Francisco et al. built an SCDT for urban energy management and leveraged smart meter electricity data to refine the interval of energy benchmarking from a year to a day^19^. In addition, it is critical to build predictive functionalities in SCDTs using the accumulated data streams, which enables proactive decision making^24^.

### Computer vision and human behavior detection

Integrating human behaviors is an important consideration in SCDT research. Computer vision techniques could be used to extract information about human behaviors from street images and video streams, making them fundamental technologies for understanding human-infrastructure interaction in cities. For example, a study by Zhang et al. used deep convolutional neural networks to analyze traffic flow in street view images, in order to model the spatio-temporal patterns of urban traffic^25^. Naik et al. used a geometric layout algorithm to analyze street view images in order to quantitatively measure the degree of change in the built environment^26^. Some studies have managed to identify and track pedestrian movement from videos rather than images. For example, Zaki et al. developed a method to identify and classify pedestrian violations in order to mitigate the risk of collisions at urban intersections^27^. Following the similar idea, they also used an object-tracking algorithm to calculate the frequency of pedestrian-vehicle collisions^28^.

Some computer vision research supports real-time multi-target (e.g., pedestrians and vehicles) detection and tracking based on images or video, which is more in line with the vision for SCDT. One of the most commonly used algorithms for real-time object detection is “You Look Only Once” (YOLO)^29^. Unlike other commonly used algorithms, such as Fast R-CNN^30^ and Mask R-CNN^31^, which include a separate region proposal step, YOLO omits this step and obtains a four-fold speedup. The Deep SORT algorithm^32^ can be combined with the YOLO together, enabling the functionality of counting pedestrians and vehicles^33^. As demonstrated in an applied study, Barthélemy et al. used YOLO for real-time detection of motion trajectories in crowds^34^.

### Urban heat exposure assessment

Heat exposure assessment has been extensively studied at the national, regional, or city levels, with a variety of spatial resolutions (shown in Table 1). The unit of analysis includes regions (e.g., south, northeast, etc.), cities, spatial units (e.g., census tracts, land use units), cells (e.g., 5 km by 5 km square), buildings, and specific locations (e.g., coordinates). For example, considering the macro spatial scale, Wang et al. assessed the heat exposure across 31 major Chinese cities, adopting over 1.544 million Weibo (the Chinese equivalent of Twitter) posts and meteorological conditions^35^. In a meso-scale assessment, Park et al. investigated the spatial patterns of deadly heat exposure for elderly populations in Seoul and Tokyo at a resolution of 1 by 1 km cells^10^. Some research efforts have examined microscopic heat exposure assessment. Yin et al. conducted a heat exposure assessment with a resolution of 30 × 30 m cells^7^. This micro-scale assessment relied on large-scale smartphone location data and temperature data collected by vehicle-mounted sensors. Li assessed heat exposure using one-square-meter cells by employing a high-resolution urban 3-dimensional digital surface model^12^. While some studies have conducted heat exposure assessments at higher resolutions (i.e., individual level), their analyses were based on individual physiological and psychological data without meteorological information input^36,37^.

**Table 1:** Comparison of recent heat exposure assessment studies in terms of spatial and temporal resolutions.

Source	Spatial resolution							Temporal resolution						Data source
	Region	City	Unit	Cell	Building	Location	Individual	Static	Year	Month	Day	Hour	Minute	
^40^					√			√						Meteorology, Census, Simulation
^38^			√					√						Meteorology, Mortality
^39^				√								√		Meteorology, Census
^36^							√	√						Wearable device
^9^	√									√				Meteorology
^2^		√									√			Meteorology, Census
^12^						√						√		Meteorology, Census, GIS
^7^						√							√	Meteorology, Cell phone
^10^				√					√					Meteorology, Census
^11^			√						√					Meteorology, Census
^37^							√	√						Survey
^35^		√									√			Meteorology, Social media
This study						√						√		Meteorology, Computer vision, Simulation

Previous studies also vary in temporal resolution. Most studies consider the heat exposure in studied areas as static or changing from year to year. Willers et al. investigated neighborhood heterogeneity in mortality rates directly related to extreme heat and air pollution in a city, based on a 15-year dataset^38^. In another example, Hua et al. developed a quantitative index that considers heat-related hazards, exposures, and vulnerabilities^11^. The index was used to assess the risk of extreme heat exposure in Hong Kong for three selected years. Some studies have calculated heat exposures in months or days. Wang et al. compared the daily maximum temperature with the daily sentiment to heat on social media to reveal their correlations^35^. Heat exposure also fluctuates within a day, and assessments in hours or even minutes can provide further information for mitigation measures. Hu et al. calculated the heat exposure by hours in the Chicago metropolitan area and found that the impact of extreme heat events varies in the temporal dimension^39^.

As shown in Table 1, studies attempting high-resolution and micro-scale spatial or temporal collective heat exposure assessments and mitigation are limited. Among the limited number of relevant studies, thermal process simulation^12,40^ is the most frequently used technique for generating micro-scale meteorological data in the spatial dimension. To collect meteorological data with small temporal intervals, satellite observation techniques were used by Hu et al.^39^. In addition, for collecting passersby counts and calculating collective heat exposure, high-resolution smartphone location data was used to obtain population movement information^7^. While previous research has made advancements in this area, the technical approaches based on simulation, remote sensing, crowdsourced data, etc., have difficulty obtaining both high temporal resolution and high spatial resolution data. In the prior work closest to this study^7^, even though crowdsourced data on human mobility could provide specific times and locations, only a subset of the population participated in the crowdsourcing. Vehicle-mounted meteorological sensors were used in that study, which are difficult to run consistently and in a real-time manner. Because of these shortcomings, previous methods have struggled to adequately support location-based and dynamically updating heat exposure mitigation measures, such as high-resolution heat exposure alerts or the establishment of cooling stations, fans, etc. Therefore, a method that can continuously assess collective heat exposure with high spatiotemporal resolution in real-time and support related decision-making is lacking.

## Methods

**Figure 1:**
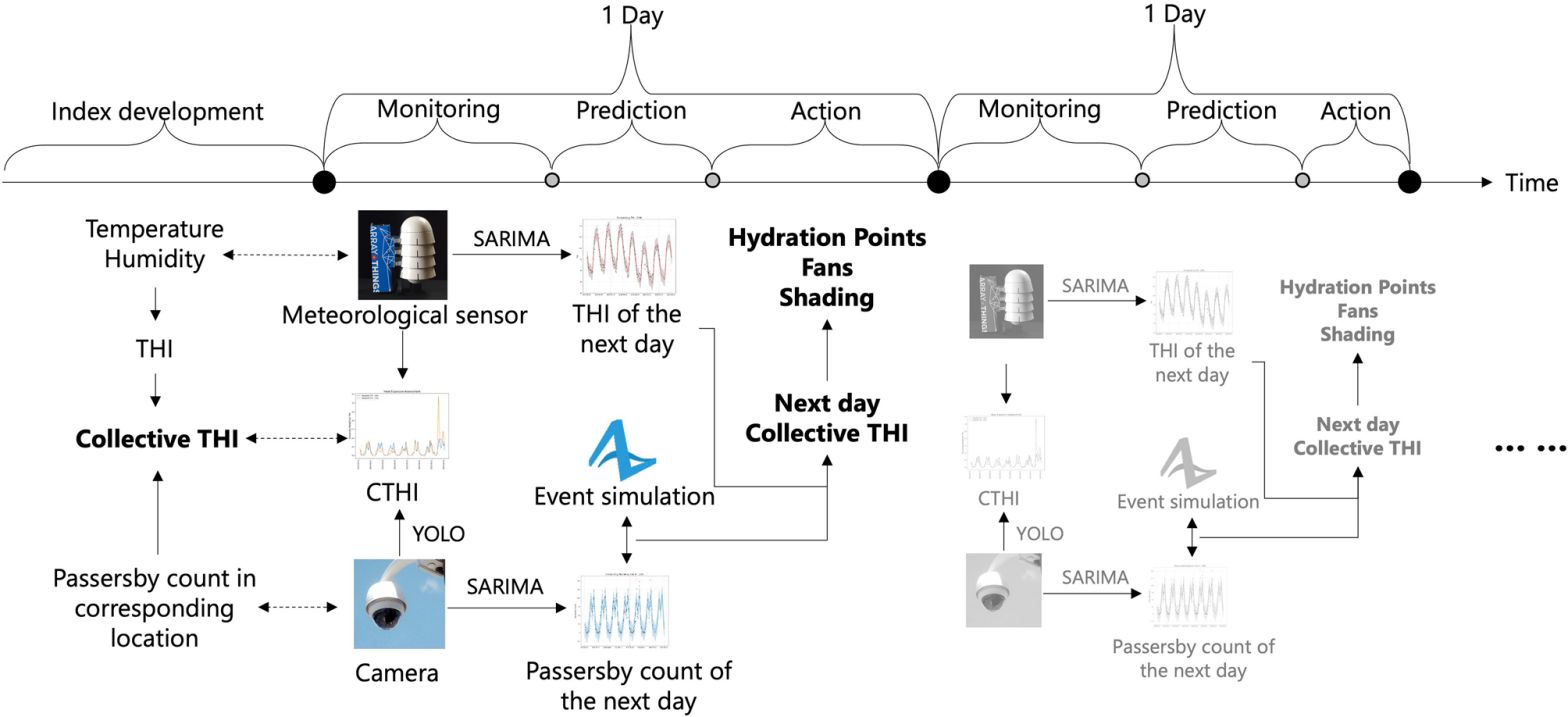
The framework of the collective urban heat exposure assessment SCDT.

Considering the shortcomings of previous studies, we have developed a novel SCDT framework for urban collective heat exposure assessment and mitigation (Fig. 1). Meteorological sensors and street cameras that can operate continuously throughout the day are used as the data collection method. They consistently collect temperature, humidity, and the number of passersby at the location in real-time, enabling a high temporal resolution collective heat exposure assessment. Along with ubiquitous sensors and cameras, this technological approach can support high spatial resolution collective heat exposure assessment. Furthermore, heat exposure should be assessed to support decision-making. Therefore, we have used a time-series forecasting approach to predict heat exposure levels based on the accumulated data to inform the possibility of extreme heat exposures and to enable proactive interventions.

The first step of the proposed method is to define the heat exposure metric. Conceptually, heat exposure is the physical strain that a person feels as a result of increasing heat storage in the body. It is influenced simultaneously by the intensity of environmental (e.g., temperature and humidity), physiological (e.g., metabolism), and behavioral (e.g., clothing) factors, as well as by the duration of that intensity. In this study, we employed temperature and humidity to measure the intensity of heat exposure since they are major environmental factors, and environmental factors can influence physiological and behavioral factors. Humidity is highlighted here, as there is a consistent and linear negative correlation between the humidity and the efficacy of perspiration evaporation, which affects apparent temperature and humidity levels. When atmospheric moisture increases, the evaporation rate from the body decreases due to the reduced capacity of the air to further absorb water vapor^41^. Therefore, the Temperature-Humidity Index (THI) is used for heat exposure assessment^42^. We used the empirical ratings from previous studies to define metric levels, ranging from “very warm” to “extremely hot”. The equation for THI and the corresponding definitions of levels are as below.

1 $$THI=t-\left(0.55-0.0055\times RH\right)\left(t-58\right)$$

where *RH* is relative humidity (%) and *t* is the temperature in degrees Fahrenheit.

The THI can measure heat exposure at a location, but the use of the THI in isolation ignores the effect of crowds on collective heat exposure and corresponding heat exposure mitigation decisions. Intuitively, the number of passersby will directly affect how important heat exposure is to the decision-making. Thus, in this study, the term ‘heat exposure' has been expanded'collective heat exposure' at a specific location, encompassing the cumulative heat exposures of all individuals at the location. Correspondingly, THI has been expanded to 'collective heat stress index' (CTHI), combining meteorological heat stress measurements with the number of passersby affected by heat exposure at a given location. Assuming the time period of heat exposure assessment is *t* (*t* = 1, 2, …, *T*) the CTHI at location *i* (*i* = 1,2, … , *I*) during time period *t* is calculated as follows:

2 $${CTHI}_{t, i}={THI}_{t,i}\times {P}_{t,i}$$

where ${P}_{t,i}$ and ${THI}_{t,i}$ indicate the passersby count and the THI value at location *i* at the time period *t*, respectively.

Real-time data streams are utilized to continuously calculate the proposed CTHI. There are two necessary data streams, one of which is the temperature and humidity of the location being studied. An open-source sensor platform, developed by the Argonne National Laboratory, known as the Array of Things (AoT), was utilized in this study^43^. Sensor nodes were mounted on roadside poles. After installation, the sensor nodes continuously collected temperature and humidity data every 30 s, which was then uploaded to the Argonne National Laboratory’s cloud server. The data stream was then downloaded from the server. Another important data stream is the passersby count. Street cameras were installed at the case study locations, providing video streams on a continuous basis. The video stream was preprocessed using YOLOv3, with parameters defined by a pre-trained model. YOLOv3 was implemented in the Python environment using OpenCV and TensorFlow libraries. Note that YOLOv3 is not able to track and match individuals between frames of a video. Therefore, the real-time object tracking algorithm DeepSORT was integrated with YOLOv3. The data streams of temperature, humidity, and passersby count were resampled using the same time interval (e.g., hourly) and were then sent to Eqs. (1) - (2) to generate the CTHI data stream.

Unlike long-period and large-area heat exposure assessments, high-resolution heat exposure indices, especially collective heat exposure indices that account for human behaviors, exhibit greater variance. The heat exposure level at the target time of implementing mitigation measures may significantly differ from that observed over the past few hours or days. Monitoring CTHI alone will not enable city managers to take proactive measures before high heat exposure occurs. Therefore, further CTHI predictions were made based on accumulated data from CTHI monitoring. CTHI prediction was divided into two parts: regular prediction and “black swan” analysis. In regular prediction, THI and passersby count are predicted using SARIMA models separately. The input to the SARIMA models is THI or passerby counts at previous time points, with the output being THI or passersby counts at the next time point. The SARIMA model requires the definition of several hyperparameters, including lag order, degree of difference, moving average order, corresponding parameters for the seasonal component and seasonal interval. The auto-ARIMA algorithm in the *pmdarima* Python package was used to estimate these hyperparameters. The estimated hyperparameters were further manually adjusted to reduce the model training loss, measured by R-square. The hyperparameters of the SARIMA models are listed in the figure captions in the Results section. Both THI and passersby count predictions were evaluated using August 22nd, 2022, for testing model performance and data from previous days for training the models. Note that the passersby count prediction was trained using data from August 16th–22nd because of limited data sources, while THI prediction utilized data for the entire month of August.

Despite being a powerful prediction approach, the SARIMA model, as well as many other machine learning tools, cannot predict “black swan” events very well. The “black swan” events are rare and unexpected occurrences that cannot be well predicted by human judgement or statistical models^44^. In this study, examples of “black swan” events include marches, protests, rallies, and other irregular community activities. These events can dramatically change the movement patterns of urban populations, affecting CTHI values, impacting collective heat exposure, and influencing decision-making for heat exposure mitigations. Thus, as the second part of the CTHI prediction methodology, crowd simulation was used to obtain the passersby count based on key information about the event. Inputs to the model included information about the urban space and infrastructure (e.g., roads, buildings, and parking lots) within the study area, as well as fundamental information about the large-scale event, such as the expected number of participants, start time, and approximate route. Agents representing the number of participants were generated in Anylogic and they were set to move along the road following the event route. The process of crowd gathering and evacuation was also modeled, including the movement of some agents to and from parking lots. The number of people passing by the study location in the simulation was recorded. The simulated passersby count was then combined with the number of people predicted by SARIMA. The adjusted passersby count was subsequently used in the calculation of CTHI.

## Results

**Figure 2:**
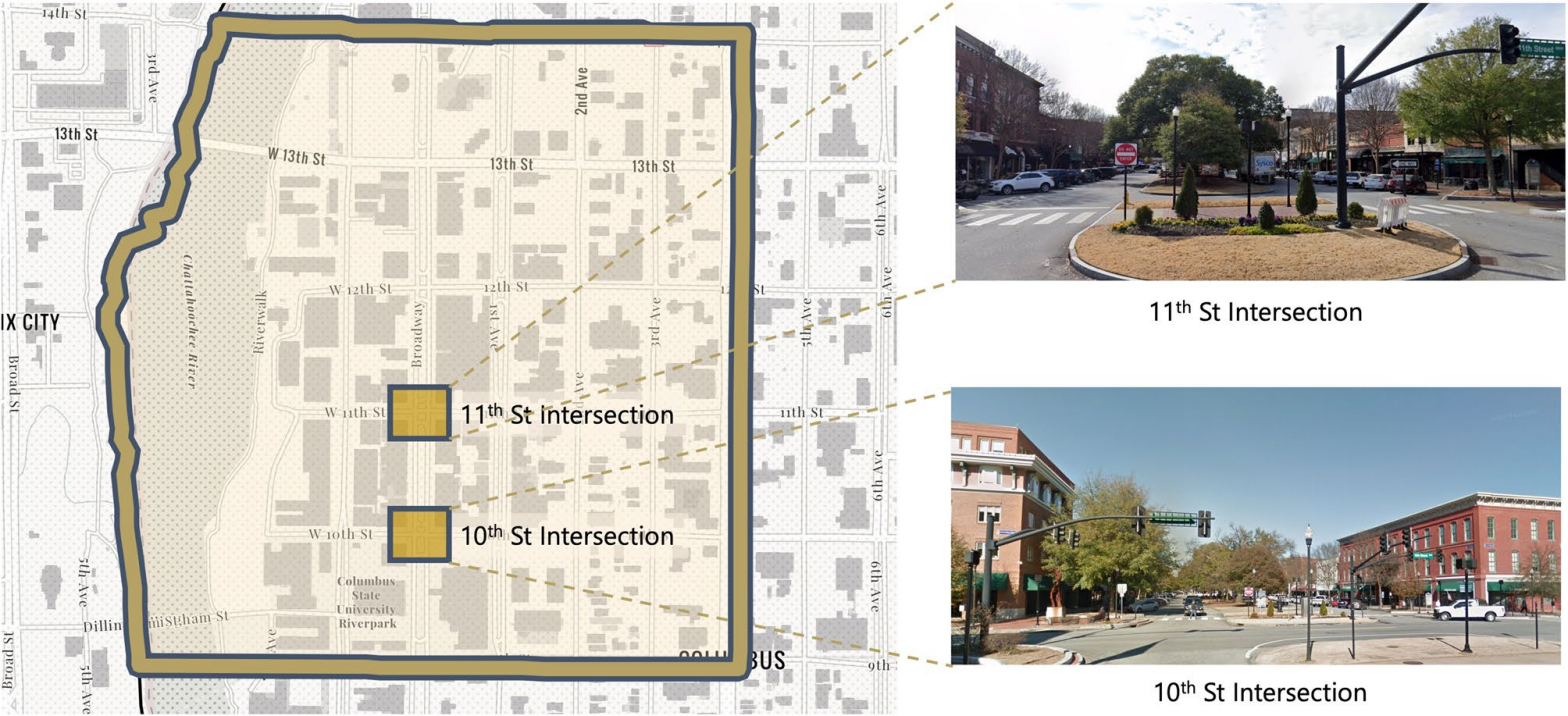
Selected case study sites, intersections of Broadway and the 10th Street and 11th Street, Columbus, GA.

A pilot experiment was conducted in Columbus, Georgia to test and demonstrate how the proposed method can contribute to the assessment, prediction, and mitigation of collective urban heat exposure. The Columbus Health Department identifies heat exposure to be a major concern for city management, as heat stroke is one of the leading causes of child mortality during heat waves. They aim to raise awareness among citizens about the severity of this problem. Assessing citizens’ heat exposure helps the Columbus Health Department to achieve this goal. Therefore, in collaboration with the the local government, two intersections in Uptown Columbus (namely, the intersections of Broadway with 10th St. and 11th St.) were selected as case study sites. Both are major intersections in the city’s business district (as shown in Fig. 2). Passersby data from Sunday, August 16, 2020, to Saturday, August 22, 2020, representing a typical summer week, were collected to evaluate the proposed methodology.

**Figure 3:**
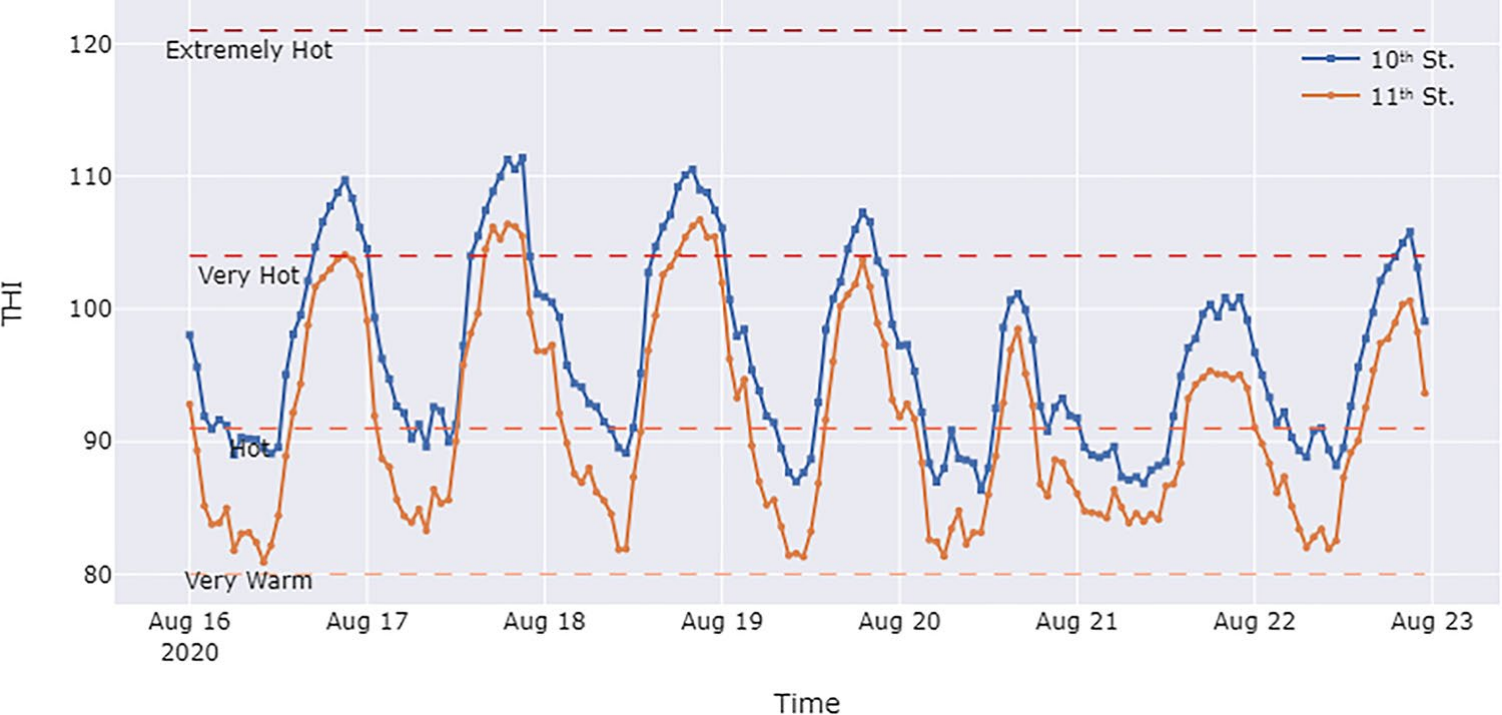
THI values between August 16th–22nd, 2020 for the two intersections in the case study.

Temperature and humidity data at the intersection of Broadway with 10th St. and 11th St. were collected by meteorological sensors mounted on the poles. These data streams were fed into Eq. 1 to generate the THI data streams. Figure 3 shows the THI values for these two intersections during the study week. It shows that the THI values at both intersections exceeded the “hot” threshold during the day and, in some cases, even reached “very hot” levels. Based on the definition of heat exposure levels in Table 2, these conditions could result in heat cramps and heat exhaustion, with a risk of heat stroke. It is important to note that the THI at the 10th Street intersection was higher than that at the 11th Street intersection. Specifically, the 10th Street intersection experienced “very hot” THIs for five days of the week, while the 11th Street intersection experienced “very hot” conditions on only two days. In addition, even at night, the THI at 10th Street remained far above the “very warm” level and approached the “hot” level for the first few days. It is also worth noting that on Sunday, August 16, between 9:00 and 10:00 p.m., the THI at the 10th Street intersection reached 108, while the 11th Street intersection had a THI of 85. This is the largest difference in THI between the two intersections, which supports the heterogeneity of heat exposure at a micro-scale and emphasizes the need for a high-resolution heat exposure assessment.

**Table 2:** Temperature-humidity index (THI) levels.

Level	THI (℉)	Effect on the body
Very warm	> 80	Prolonged exposure and activities may lead to fatigue, which may be severe enough to cause heat cramps.
Hot	> 90	May lead to heat cramps and heat exhaustion, and in severe cases may lead to heat stroke.
Very hot	> 105	The risk of heat cramps and heat exhaustion is very high, and heat stroke is probable in severe cases.
Extremely hot	> 128	Heat stroke risk is high.

**Figure 4:**
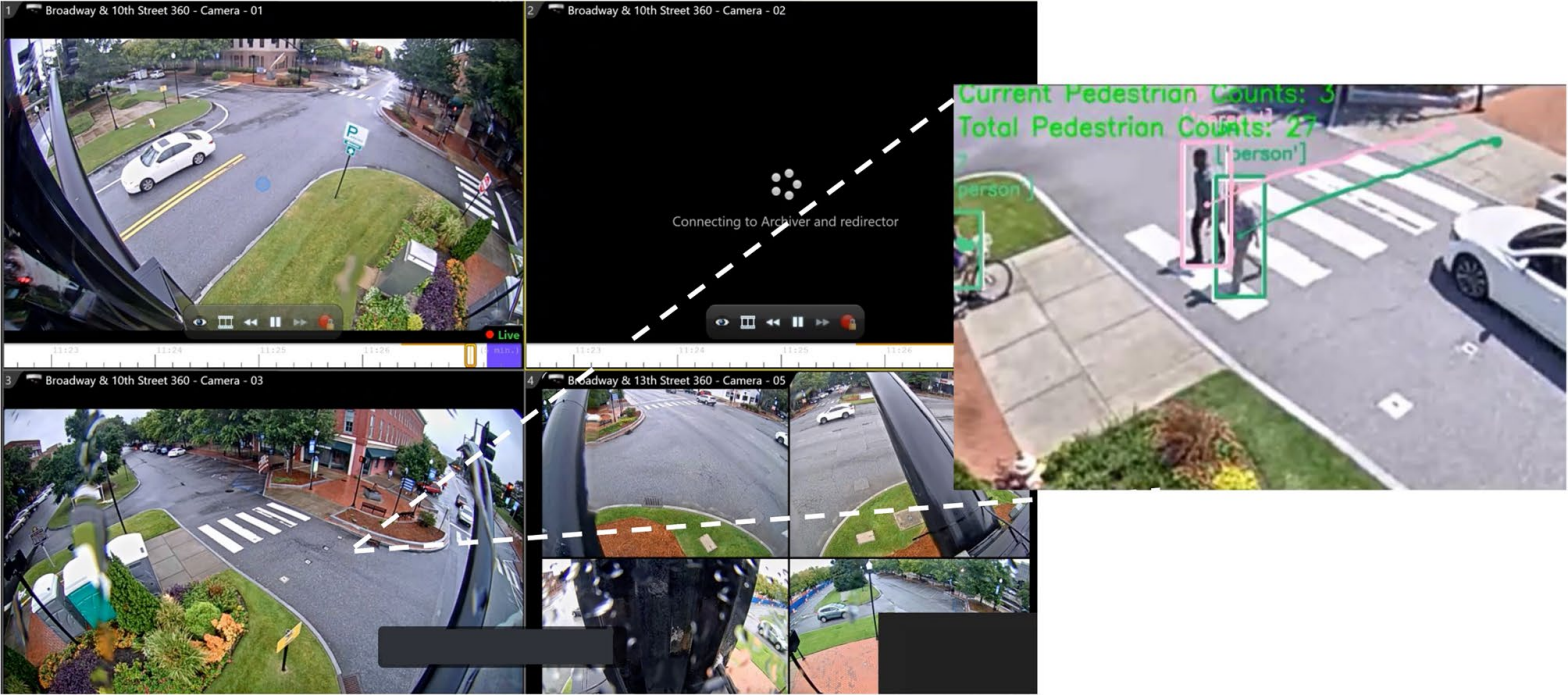
Street videos collected by cameras undergo passersby analysis by the YOLO and Deep SORT algorithm.

**Figure 5:**
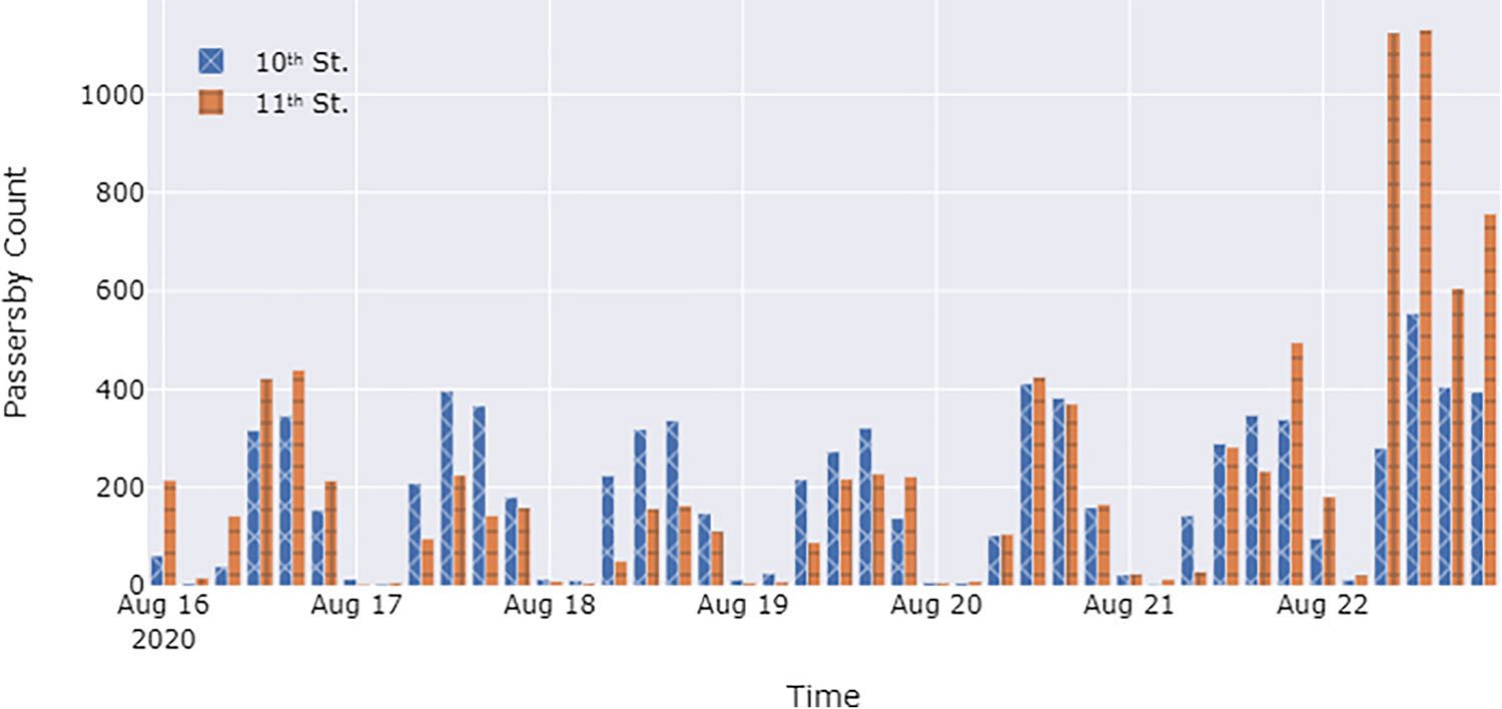
Passersby count per four hours between Aug 16th–22nd, 2020 for the two intersections in the case study.

The passersby count data are derived from the analysis of street video streams (Fig. 4). The videos are analyzed by the YOLOv3 and DeepSORT algorithms, as described in the Methods section, which derive the passersby count for each time point at the specific location (shown in Fig. 5). Under normal conditions, no more than 400 passersby pass through each intersection every four hours. In the studied week, the weekday passersby count at the 10th Street intersection was similar to, and sometimes higher than, that at the 11th Street intersection. However, there was an organized Black Lives Matter protest on the 11th Street on August 22nd. The number of people passing through the 11th Street intersection increased dramatically that day. The number of people passing through the 11th Street intersection at the peak reached more than 1,000 people per four hours. From 12:00 a.m. to 4:00 p.m., 1,624 pedestrians crossed the 11th Street intersection, while only 425 pedestrians crossed the 10th Street intersection.

**Figure 6:**
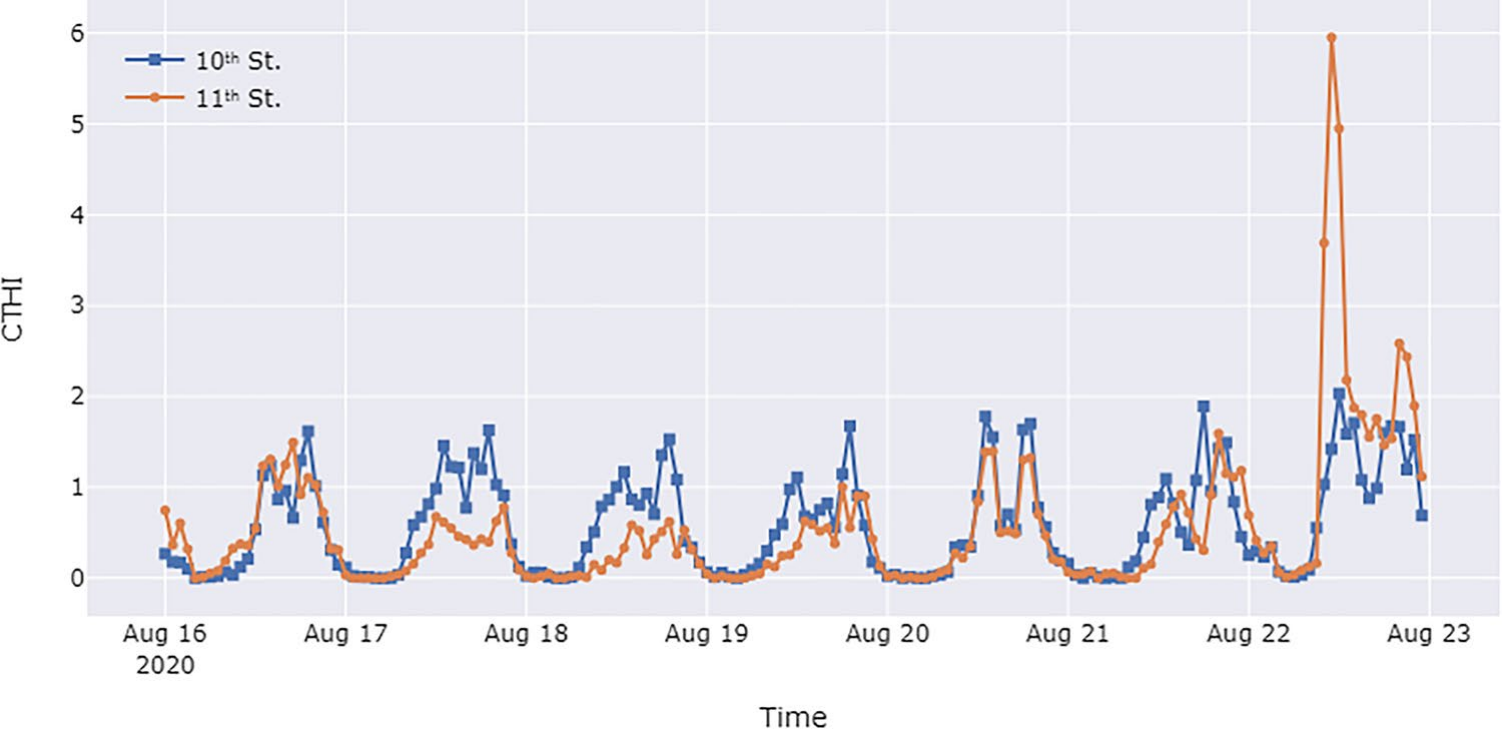
CTHI between August 16th–22nd, 2020 for the two intersections in the case study.

The meteorological data collected by the AoTs and the passersby data were combined to calculate the CTHI according to Eq. 2. Figure 6 plots the CTHI variation for the studied week. Compared to THI, CTHI exhibits different heat exposure curves. For example, Fig. 3 shows that the THI at the 10th Street intersection was significantly higher than that at the 11th Street intersection on August 16th, which indicates that 10th Street is prone to higher heat exposure than 11th Street. However, Fig. 6 demonstrates that on August 16, the CTHI at 11th Street was higher than that at 10th Street for most of the day. This difference is attributable to the fact that more passersby crossed the 11th Street intersection on the day (as shown in Fig. 5), resulting in an increase in collective heat exposure. Another example illustrated in Fig. 6 and the CTHI data is the spike in heat exposure caused by the protests on August 22nd, but Fig. 3 and the THI data do not show this phenomenon.

**Figure 7:**
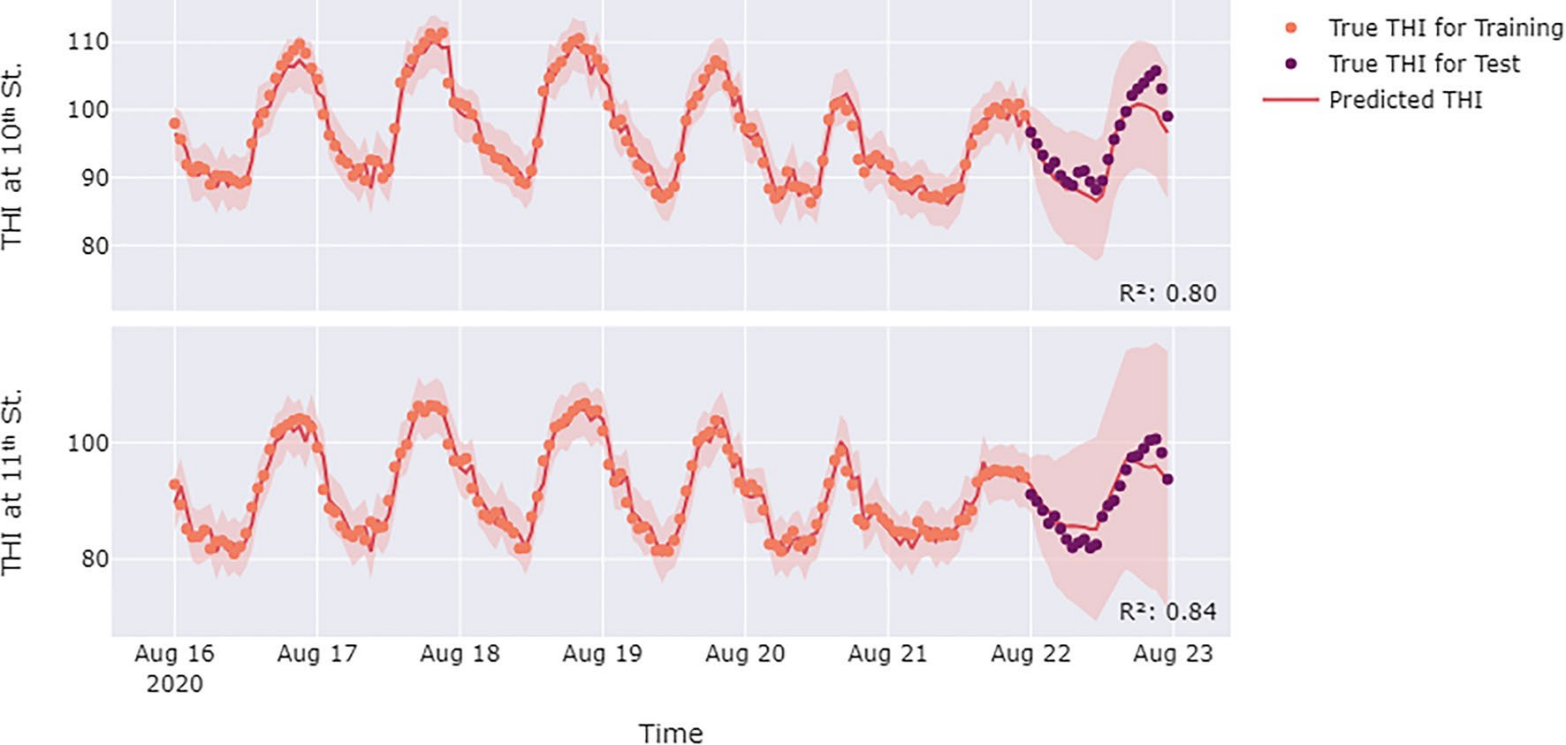
Predictions of the THI on August 22nd at the 10th and 11th Street intersections. The SARIMA model for the 10th Street intersection was defined by (2, 0, 2)(4, 0, 1)24, and the SARIMA for 11th Street intersection was defined by (3, 1, 0)(5, 0, 0)24. The R^2^ value in the figure indicates the prediction accuracy for August 22nd.

**Figure 8:**
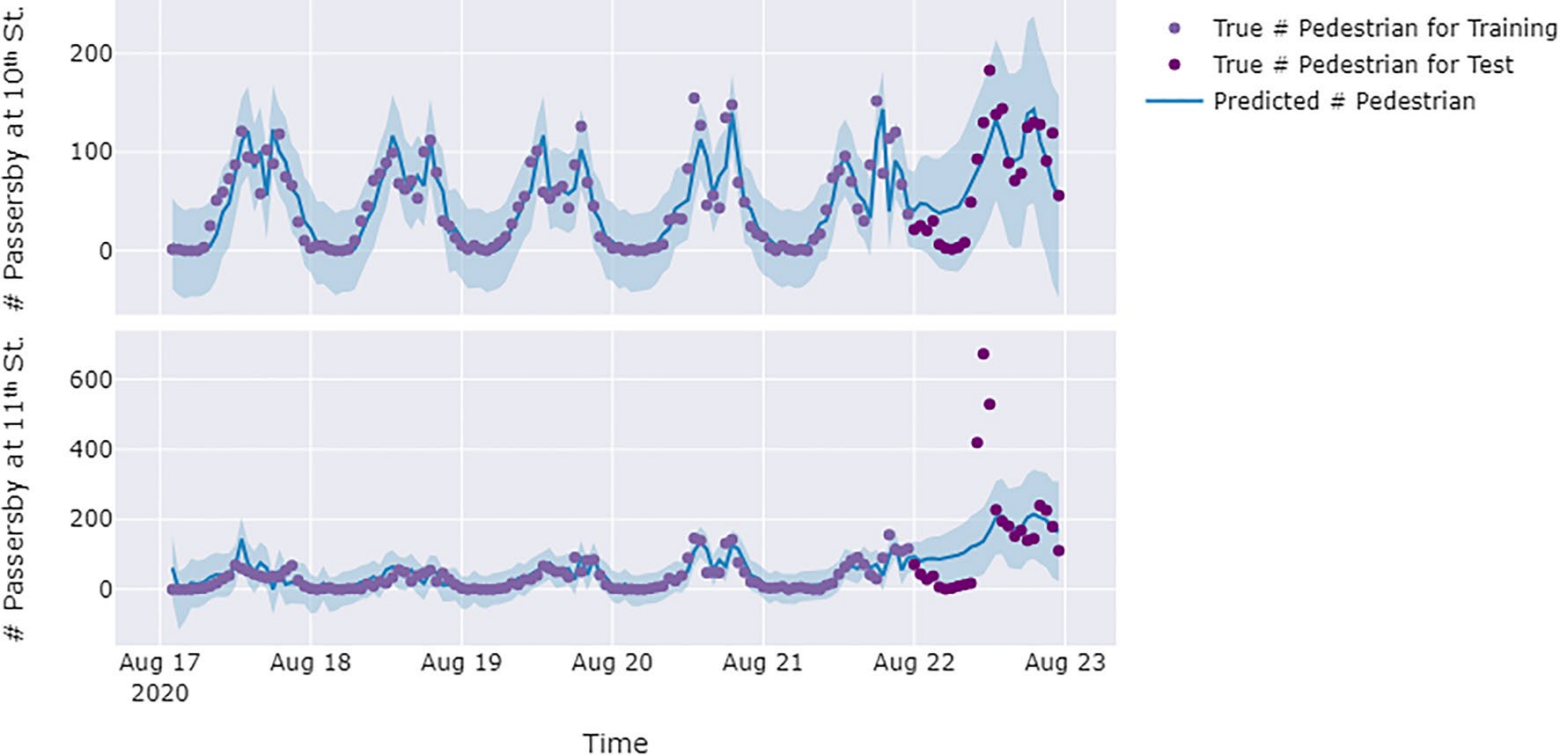
Predictions of the passersby count on August 22nd at the 10th and 11th Street intersections. The SARIMA model for 10th Street was defined by (4, 1, 0)(3, 0, 1)24, and the SARIMA for 11th Street was defined by (4, 2, 3)(2, 1, 1)24. The R^2^ value in the figure indicates the prediction accuracy for August 22nd.

Heat exposure monitoring enables the accumulation of meteorological and behavioral data from the studied locations, which facilitates short-term predictions of future collective heat exposure. For this purpose, we employed SARIMA to predict collective heat exposure. Specifically, we predicted the THI and passersby count of August 22nd in normal conditions using data from the first six days of the week. Figures 7 and 8 present the forecasting of THI and passersby count for the intersections on 10th and 11th Street. The black dots represent the real data points, the blue or red lines are the trained model, and the shaded areas constitute the upper and lower confidence bounds of the model. As depicted in Fig. 7 and 8, the predictions maintain a similar daily variation trend as observed thoughout the week, while being influenced by day-to-day variation trends.

As mentioned in the Methods section, the SARIMA model is limited in predicting “black swan” events, and the data accumulated in SCDT may not be sufficient to predict the spatial and temporal distribution patterns of crowds during large and rare events, such as protests. Therefore, we employed agent-based simulation to improve the prediction performance for such less predictable events. Users of the SCDT can input fundamental information about the August 22nd scheduled protest into the simulation model. This includes details such as the approximate number of participants, the start time of the protest, and potential routes. The input parameters would enable “what-if*”* scenario analyses. A crowd simulation model can then be constructed for each scenario and be used to obtain the specific spatiotemporal distribution of the crowd during the protest.

**Figure 9:**
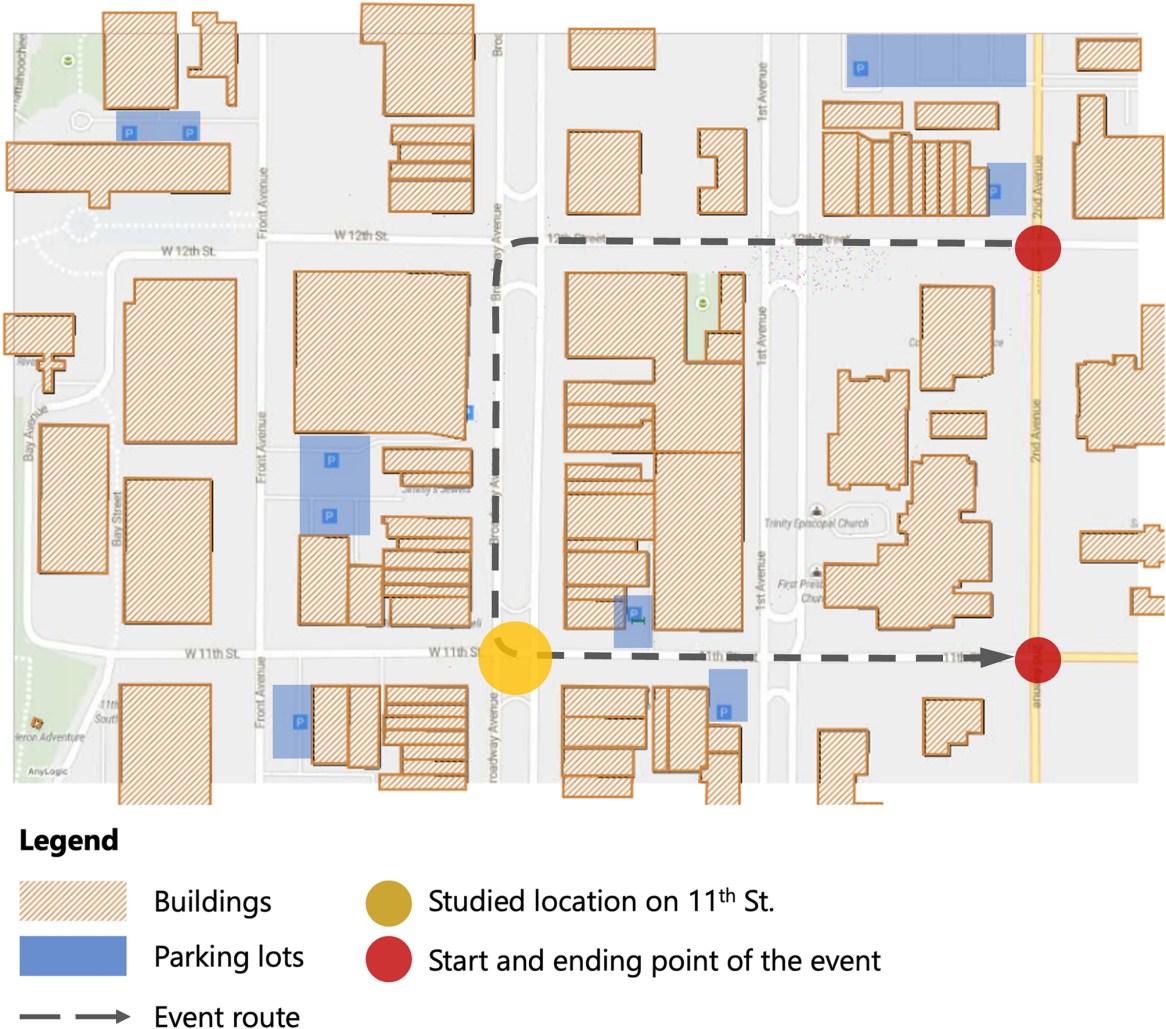
Agent-based simulation settings for predicting the spatiotemporal distribution of event participants.

Figure 9 shows the spatial configuration of the event on August 22nd in Anylogic simulation model. The agents’ motion environment relied on Anylogic’s Geographical Information System Map, which supports the insertion of real city maps. The buildings where the event was held were imported into Anylogic via a Shapefile file obtained from Geofabrik and worked as barriers to agents. Because the event does not pass through the intersection on 10th St., this intersection is not included in the simulation. It was assumed that the protest participants would drive to Uptown Columbus, park in a nearby parking lot, walk to the assembly area (i.e., start point), and wait for the event to begin. The event begins at 11:00 a.m., with the crowd assembling at 12th Street, proceeding through the intersection of 11th Street and Broadway, and then down 11th Street to 2nd Avenue. The crowd then disperses, returns to the parking lot, and drives away. Five hundred agents were created in the simulation, and these agents arrived gradually within a 1-hour timeframe. The movement velocity of the agents was set to a uniform distribution between 0.5 and 1 m per second. The number of passersby crossing the 11th Street intersection was recorded in the simulation model as output. The output is the average of 10 runs of the simulation model. This output was then added to the passersby count predicted by SARIMA to obtain the passersby count prediction in the “black swan” event and the resulting CTHI.

**Figure 10:**
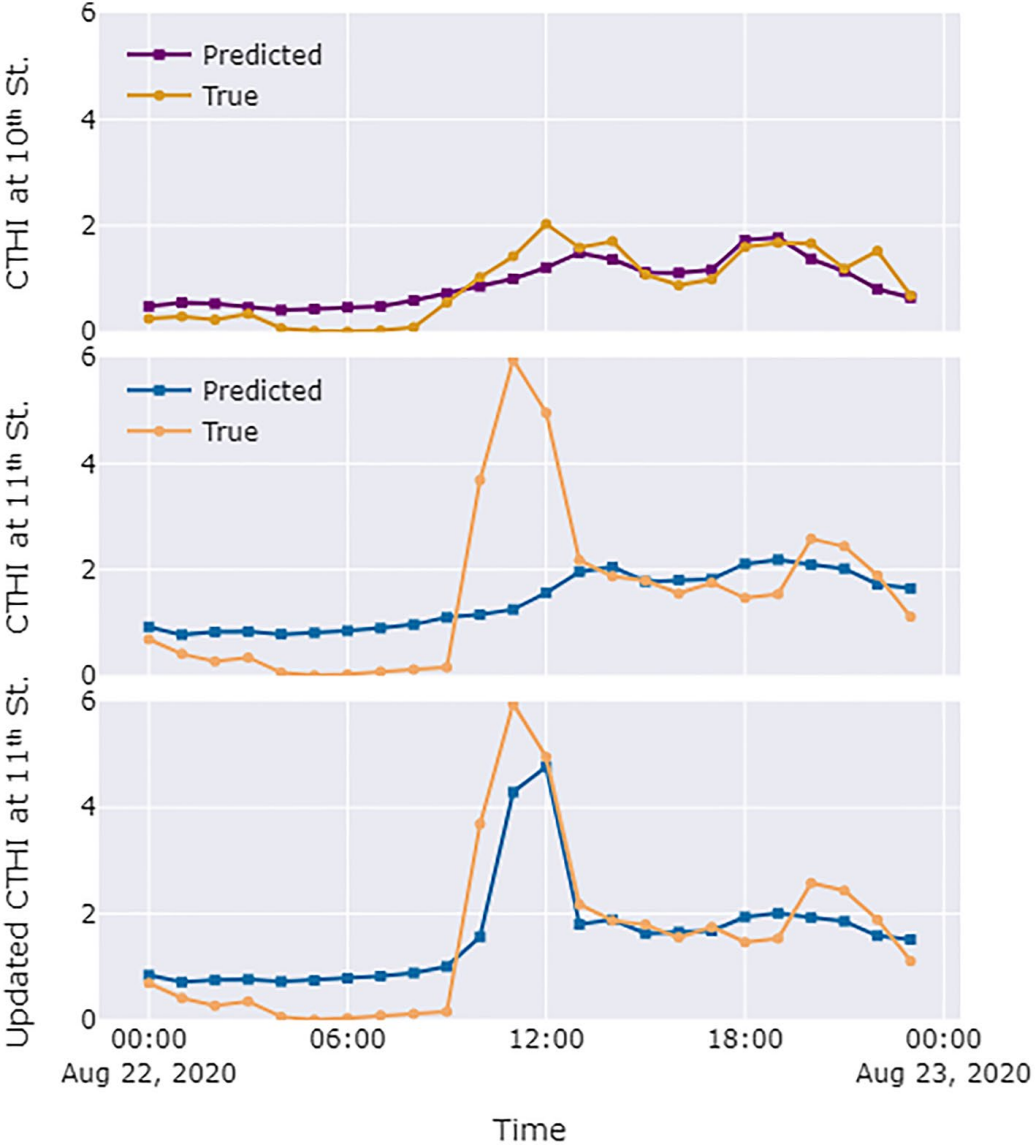
Prediction of the CTHI at the 10th Street intersection, the 11th Street intersection, and the updated CTHI by using the results from the simulation at the 11th Street intersection.

The first plot in Fig. 10 evaluates the prediction for collective heat exposure at the 10th Street intersection on August 22nd. It can be seen that the predicted and actual heat exposures have similar temporal trends. In addition, although the predicted heat exposure at noon was lower than the actual heat exposure, the predicted and the actual values were close for the rest of the day. The two plots below show the 11th Street intersection CTHI predictions before and after updating the estimation by using the simulation. Before the update, it is obvious that the predicted values of CTHI are significantly lower than the ground truth during the event period. This is attributed to the fact that the historical data used by the SARIMA model does not contain large and rare events. After incorporating the simulation, the CTHI rises significantly between 11:00 a.m.and 3:00 p.m. and approaches the true value. Based on the predictions in Fig. 10, city managers can obtain more information about the potential extreme heat exposure events in advance, such as a more specific time of the peak heat exposure, and take heat mitigation measures proactively (e.g., deploying hydration stations, fans, and/or shading).

## Discussion

In this study, we propose a novel SCDT framework for urban heat exposure mitigation with high-resolution collective assessment. In order to support the implementation of heat exposure mitigation measures, the importance of measures targeting different times and locations needs to be assessed. Because an increase in the passersby count raises the importance of collective heat exposure and the adoption of corresponding measures, the conventional THI was expanded to CTHI. To further provide the specific time and location of important measures, meteorological sensors and street cameras were installed in multiple locations to provide high temporal and spatial resolution CTHI in real-time. As the final step, to enable taking actions prior to the occurrence of extreme heat exposures, the CTHI was predicted by using time series forecasting and simulation methods.

### Contributions

The first part of the proposed framework involves monitoring the collective heat exposure at fine-grained spatial locations using AoT sensor nodes and the YOLOv3 and DeepSORT algorithms. Compared with prior studies^2,9,11,39^, the proposed framework in this paper offers several advancements in heat exposure mitigation decision-making. First, the proposed framework can reveal more variations in heat exposure across spatiotemporal dimensions. As shown in Fig. 6, despite the proximity of the 10th Street and the 11th Street intersections, the collective heat exposures are clearly different on August 17 and 18, with the CTHI at 10th Street much higher than that at 11th Street. In addition, although the trends of heat exposures at the two intersections are similar on August 21st, the heat exposure at the 10th Street intersection is higher than that on the 11th Street intersection at noon and in the late afternoon, but lower in the afternoon and evening. This heat exposure information offers high spatiotemporal resolution and is therefore valuable for heat exposure mitigation decisions. Based on this information, city managers can be more agile in establishing and locating heat exposure mitigation facilities, such as hydration stations, fans, and shading, or more accurately informing citizens of heat exposure risks.

Second, the proposed framework integrates meteorological information with collective human movements and improves the accuracy of heat exposure assessment by using the CTHI. It is shown in Fig. 3 that the THI at the 10th Street intersection is clearly higher than that at the 11th Street intersection on August 16. However, the results of CTHI are clearly different. As shown in Fig. 6, the CTHI at the 11th Street intersection is actually higher than that at the 10th Street intersection, as there are more people passing by the 11th Street intersection. By taking into account human movement and the consequent exposure, the proposed framework can mitigate, to some extent, the risk of underestimating or overestimating the impact of heat exposure on citizens, thereby facilitating more precise and informed decision-making.

Although Yin et al. also designed new metrics to integrate meteorological states and human movement, their work focused solely on heat exposure assessment and did not include efforts to estimate and proactively address future collective heat exposure^7^. The second component of the proposed SCDT framework uses a SARIMA model and the data accumulated from heat exposure monitoring to predict the future THI, passersby count, and CTHI. In particular, a crowd simulation model was used as a complementary tool to predict the passersby count during large and rare social events. The proposed comprehensive framework enables proactive actions to mitigate heat exposures. Based on the predicted CTHI, decision-makers can prioritize locations that have both high heat exposure risk and a large number of passersby and deploy heat exposure mitigation resources accordingly. 

### Limitations and Future Work

Although this study has made significant contributions, there are still some limitations to address in future research. First, the heat exposure assessment in this study primarily focuses on temporal heat exposure intensities for multiple specific locations. While this approach is favorable for taking location-specific heat exposure mitigation measures, a human-specific heat exposure assessment needs to consider the duration of exposure with each intensity. An example is that the intensity of an individual’s heat exposure varies over its movement trajectory. For another example, at the same location, some individuals may have just come out from indoors and have a low overall heat exposure, while others may have been walking in the city for some time and have a higher heat exposure. The proposed method is unable to track an individual's movement trajectory and the duration at each heat exposure intensity.

Second, heat exposure assessment methods should consider both environmental and social factors. This study includes two social factors, passersby count and an infrequent social event, as part of the model inputs. However, due to the limited availability of data, the consideration of social factors an be improved. For example, when exceeding a threshold, the high density of the crowd may influence the micro-scale environment, such as wind and humidity, resulting in higher heat exposure inside the crowd than outside. This effect was not modeled in this study. As another example, more features are favorable for the prediction of passersby counts. Changes in traffic and commercial operations (e.g., traffic congestion, commercial closures on holidays) around a location can lead to changes in human movement patterns, as well as changes in collective heat exposure. Third, although THI thresholds and heat exposure levels are presented in Table 2, the newly proposed CTHI thresholds were not defined in this study. Users do not know what level of heat exposure risk is implied by the value of CTHI. Fourth, the initiation of SCDT projects is often feasible in the short term, but the long-term benefits of the projects are often uncertain, as they are usually innovative solutions and lack previous reference cases. This study does not present and investigate the long-term effectiveness of the proposed SCDT system and the difficulties that may be encountered.

In response to these limitations, we propose several future research directions. First, in order to assess the heat exposure at the individual level, a computer vision method that can match the same individual across different video streams in various locations is needed. By matching individuals’ identities in different video streams, the trajectories of an individual moving in the city can be captured, which provides the duration of their stay at each location. Combined with the THIs along the trajectory, individual-specific heat exposures can be modeled, providing higher granularity information for collective heat exposure assessment and corresponding decision-making. However, potential privacy concerns would need to be addressed.

Second, in order to consider the social factors in collective heat exposure assessment more comprehensively, the increased heat exposure due to the high crowd density needs to be modeled and added to the CTHI. This goes beyond identifying and counting the passersby at a location and requires measuring the distance among passersby and their speed of movement. The thermal processes within the crowd and the empirical relationship between density and heat exposure also need to be specified. Conversely, in order to predict the number of passersby more accurately in time series prediction and simulations, the surrounding population activities, such as the real-time traffic status and the operational status of the point of interest (e.g., shopping malls, schools, etc.), need to be collected and added to the models as features. This requires establishing more data-sharing collaborations and building more real-time data streams.

Third, to establish thresholds for the CTHI, future studies can continuously collect CTHI data obtained by the proposed SCDT. By analyzing a large number of data points over time, CTHI thresholds can be defined using percentage intervals (e.g., the top 25 percent of CTHI values indicate extreme heat). Fourth, more research can focus on the project management and organizational issues brought by SCDT projects, and greater attention could be directed to the governance issues in innovative smart city projects, such as stakeholder collaboration and value distribution.

## Conclusion

In this study, we present a Smart City Digital Twin-based method for monitoring and predicting collective heat exposure. The proposed method utilizes meteorological sensors and street video streams to continuously collect data in real-time. It calculates a collective heat exposure index, predicts short-term future heat exposure, and informs heat exposure mitigation measures. With the implementation of the proposed SCDT, city managers are expected to be better equipped to recognize or foresee impending anomalies and subsequently take action to mitigate the negative impacts of heat exposure on city residents. In addition, the application and dissemination of the proposed system may enhance urban residents' awareness of heat exposure levels within the community. This heightened awareness can help encourage voluntary mitigation measures and reduce the possibility of negative health impacts from heat exposure in a timely manner.

## Data Availability

The datasets used and/or analyzed during the current study are available from the corresponding author upon reasonable request.

## References

[1] The National Institute for Occupational Safety and Health (NIOSH). Heat Stress. *Centers for Disease Control and Prevention* (2021)

[2] Zhang Y (2020). Population exposure to concurrent daytime and nighttime heatwaves in Huai River Basin. China. Sustainable Cities and Society.

[3] Vicedo-Cabrera AM (2021). The burden of heat-related mortality attributable to recent human-induced climate change. Nat. Clim. Chang..

[4] Paravantis J, Santamouris M, Cartalis C, Efthymiou C, Kontoulis N (2017). Mortality associated with high ambient temperatures, heatwaves, and the urban heat island in Athens. Greece. Sustainability.

[5] Macintyre HL, Heaviside C (2019). Potential benefits of cool roofs in reducing heat-related mortality during heatwaves in a European city. Environ. Int..

[6] United Nations Department of Economic and Social Affairs. *World Population Prospects 2019*. (2019).

[7] Yin Y (2021). DTEx: A dynamic urban thermal exposure index based on human mobility patterns. Environ. Int..

[8] Bolitho A, Miller F (2017). Heat as emergency, heat as chronic stress: policy and institutional responses to vulnerability to extreme heat. Local Environ..

[9] Li L, Zha Y (2020). Population exposure to extreme heat in China: Frequency, intensity, duration and temporal trends. Sustain. Cities Soc..

[10] Park CY, Thorne JH, Hashimoto S, Lee DK, Takahashi K (2021). Differing spatial patterns of the urban heat exposure of elderly populations in two megacities identifies alternate adaptation strategies. Sci. Total Environ..

[11] Hua J, Zhang X, Ren C, Shi Y, Lee T-C (2021). Spatiotemporal assessment of extreme heat risk for high-density cities: A case study of Hong Kong from 2006 to 2016. Sustain. Cities Soc..

[12] Li X (2021). Investigating the spatial distribution of resident’s outdoor heat exposure across neighborhoods of Philadelphia, Pennsylvania using urban microclimate modeling. Sustain. Cities Soc..

[13] Kuras ER, Hondula DM, Brown-Saracino J (2015). Heterogeneity in individually experienced temperatures (IETs) within an urban neighborhood: insights from a new approach to measuring heat exposure. Int. J. Biometeorol..

[14] Mohammadi, N. & Taylor, J. E. Smart City Digital Twins. in *2017 IEEE Symposium Series on Computational Intelligence* (2017).

[15] Mohammadi N, Taylor JE (2021). Thinking fast and slow in disaster decision-making with smart city digital twins. Nat. Comput. Sci..

[16] Hochhalter, J. *et al. Coupling Damage-Sensing Particles to the Digitial Twin Concept*. https://ntrs.nasa.gov/citations/20140006408 (2014) doi:https://ntrs.nasa.gov/search.jsp?R=20140006408.

[17] Glaessgen, E., Stargel, D. The Digital Twin Paradigm for Future NASA and U.S. Air Force Vehicles. in *53rd Structures, Structural Dynamics, and Materials Conference* (American Institute of Aeronautics and Astronautics, 2012). doi:10.2514/6.2012-1818

[18] Grieves, M. & Vickers, J. Digital Twin: Mitigating Unpredictable, Undesirable Emergent Behavior in Complex Systems. in *Transdisciplinary Perspectives on Complex Systems* 85–113 (Springer International Publishing, 2017). doi:10.1007/978-3-319-38756-7_4.

[19] Francisco A, Mohammadi N, Taylor JE (2020). Smart city digital twin-enabled energy management: toward real-time urban building energy benchmarking. J. Manag. Eng..

[20] Lin Y, Cheung W (2020). Developing WSN/BIM-based environmental monitoring management system for parking garages in smart cities. J. Manag. Eng..

[21] Fan C, Jiang Y, Mostafavi A (2020). Social sensing in disaster city digital twin: integrated textual-visual-geo framework for situational awareness during built environment disruptions. J. Manag. Eng..

[22] Lee G, Choi B, Ahn CR, Lee S (2020). Wearable biosensor and hotspot analysis-based framework to detect stress hotspots for advancing elderly’s mobility. J. Manag. Eng..

[23] Ham Y, Kim J (2020). Participatory sensing and digital twin city: updating virtual city models for enhanced risk-informed decision-making. J. Manag. Eng..

[24] Lu, Q., Parlikad, A. K., Woodall, P., Ranasinghe, G. D., Heaton, J. Developing a Dynamic Digital Twin at a Building Level: using Cambridge Campus as Case Study. in *International Conference on Smart Infrastructure and Construction 2019 (ICSIC)* vol. 36 67–75 (ICE Publishing, 2019).

[25] Zhang F, Wu L, Zhu D, Liu Y (2019). Social sensing from street-level imagery: A case study in learning spatio-temporal urban mobility patterns. ISPRS J. Photogramm. Remote. Sens..

[26] Naik N, Kominers SD, Raskar R, Glaeser EL, Hidalgo CA (2017). Computer vision uncovers predictors of physical urban change. Proc. Natl. Acad. Sci..

[27] Zaki MH, Sayed T, Ismail K, Alrukaibi F (2012). Use of computer vision to identify pedestrians’ nonconforming behavior at urban intersections. Transp. Res. Record: J. Transp. Res. Board.

[28] Zaki MH, Sayed T, Tageldin A, Hussein M (2013). Application of computer vision to diagnosis of pedestrian safety issues. Transp. Res. Record: J. Transp. Res. Board.

[29] Redmon, J., Divvala, S., Girshick, R. & Farhadi, A. You Only Look Once: Unified, Real-Time Object Detection. (2015).

[30] Girshick, R. Fast R-CNN. in *2015 IEEE International Conference on Computer Vision (ICCV)* 1440–1448 (IEEE, 2015). doi:10.1109/ICCV.2015.169

[31] He, K., Gkioxari, G., Dollar, P., Girshick, R. Mask R-CNN. in *2017 IEEE International Conference on Computer Vision (ICCV)* 2980–2988 (IEEE, 2017). doi:10.1109/ICCV.2017.322

[32] Wojke, N., Bewley, A., Paulus, D. Simple online and realtime tracking with a deep association metric. (2017)

[33] Yang, Y.-L., Gao, W.-W. A Method of Pedestrians Counting Based on Deep Learning. in *2019 3rd International Conference on Electronic Information Technology and Computer Engineering (EITCE)* 2010–2013 (IEEE, 2019). doi:10.1109/EITCE47263.2019.9094838.

[34] Barthélemy J, Verstaevel N, Forehead H, Perez P (2019). Edge-computing video analytics for real-time traffic monitoring in a smart city. Sensors.

[35] Wang J (2021). Mapping the exposure and sensitivity to heat wave events in China’s megacities. Sci. Total Environ..

[36] Runkle JD (2019). Evaluation of wearable sensors for physiologic monitoring of individually experienced temperatures in outdoor workers in southeastern US. Environ. Int..

[37] Hondula DM (2021). Novel metrics for relating personal heat exposure to social risk factors and outdoor ambient temperature. Environ. Int..

[38] Willers SM (2016). High resolution exposure modelling of heat and air pollution and the impact on mortality. Environ. Int..

[39] Hu L, Wilhelmi OV, Uejio C (2019). Assessment of heat exposure in cities: Combining the dynamics of temperature and population. Sci. Total Environ..

[40] Taylor J (2016). Mapping indoor overheating and air pollution risk modification across Great Britain: A modelling study. Build. Environ..

[41] Che Muhamed AM, Atkins K, Stannard SR, Mündel T, Thompson MW (2016). The effects of a systematic increase in relative humidity on thermoregulatory and circulatory responses during prolonged running exercise in the heat. Temperature.

[42] Steadman RG (1979). The assessment of sultriness. Part I: A temperature-humidity index based on human physiology and clothing science. J. Appl. Meteorol..

[43] Beckman, P. *et al.* Waggle: An open sensor platform for edge computing. in *2016 IEEE SENSORS* 1–3 (IEEE, 2016). doi:10.1109/ICSENS.2016.7808975.

[44] Wabartha, M., Durand, A., Francois-Lavet, V, Pineau, J. Handling black swan events in deep learning with diversely extrapolated neural networks. *IJCAI International Joint Conference on Artificial Intelligence* **2021**-**Janua**, 2140–2147 (2020)

